# Comparative morphology of the mouthparts of the megadiverse South African monkey beetles (Scarabaeidae: Hopliini): feeding adaptations and guild structure

**DOI:** 10.7717/peerj.1597

**Published:** 2016-01-21

**Authors:** Florian Karolyi, Teresa Hansal, Harald W. Krenn, Jonathan F. Colville

**Affiliations:** 1 Department of Integrative Zoology, University of Vienna, Vienna, Austria; 2 Kirstenbosh Research Center, South African National Biodiversity Institute, Cape Town, South Africa; 3 Statistic in Ecology, Environment and Conservation, Department of Statistical Science, University of Cape Town, Rondebosh, Cape Town, South Africa

**Keywords:** Flower visiting, Monkey beetles, Pollination, Guild structure, Functional morphology, Mouthparts, Hopliines

## Abstract

Although anthophilous Coleoptera are regarded to be unspecialised flower-visiting insects, monkey beetles (Scarabaeidae: Hopliini) represent one of the most important groups of pollinating insects in South Africa’s floristic hotspot of the Greater Cape Region. South African monkey beetles are known to feed on floral tissue; however, some species seem to specialise on pollen and/or nectar. The present study examined the mouthpart morphology and gut content of various hopliine species to draw conclusions on their feeding preferences. According to the specialisations of their mouthparts, the investigated species were classified into different feeding groups. Adaptations to pollen-feeding included a well-developed, toothed molar and a lobe-like, setose lacinia mobilis on the mandible as well as curled hairs or sclerotized teeth on the galea of the maxillae. Furthermore, elongated mouthparts were interpreted as adaptations for nectar feeding. Floral- and folial-tissue feeding species showed sclerotized teeth on the maxilla, but the lacinia was mostly found to be reduced to a sclerotized ledge. While species could clearly be identified as floral or folial tissue feeding, several species showed intermediate traits suggesting both pollen and nectar feeding adaptations. Mismatches found between mouthpart morphology and previously reported flower visiting behaviours across different genera and species requires alternative explanations, not necessarily associated with feeding preferences. Although detailed examinations of the mouthparts allowed conclusions about the feeding preference and flower-visiting behaviour, additional morphological and behavioural investigations, combined with greater taxon sampling and phylogenetic data, are still necessary to fully understand hopliine host plant relationships, related to monkey beetle diversity.

## Introduction

In the past decades increasing evidence has demonstrated that hundreds of plant species in various families rely on beetle pollination (Bernhardt, 2000). Recently, Wardaugh (2015) considered them one of the “big four” of insect orders that contribute the bulk of flower-visiting and pollinating insects. Flower-visiting beetle species appear to be associated with a wide range of plant species–within both gymnosperms and angiosperms–representing a diverse array of flower shapes and sizes (Dafni et al., 1990; Steiner, 1998a; Ratnayake et al., 2007; Procheş & Johnson, 2009; Steenhuisen et al., 2010). Beetles visit flowers to feed on pollen, nectar and floral tissue. However, compared to the highly adapted mouthparts of many other flower-visiting insects (Bauder, Lieskonig & Krenn, 2011; Bauder et al., 2013; Karolyi et al., 2012; Karolyi et al., 2013; Karolyi et al., 2014), anthophilous Coleoptera are generally thought to possess rather unspecialised biting-chewing mouthparts (Krenn, Plant & Szucsich, 2005). Although several past studies refer to the feeding behaviour of flower-visiting beetles (Handschin, 1929; Schremmer, 1961; Schicha, 1967; Fuchs, 1974), detailed morphological studies of mouthparts in anthophilous Coleoptera are generally rare. Some studies, however, have shown that specialised mouthparts that exhibit particular adaptations to feed on pollen and/or nectar are present across different beetle taxa (Schicha, 1967; Johnson & Nicolson, 2001; Karolyi, Gorb & Krenn, 2009; Wilhelmi & Krenn, 2012). Morphological adaptations and specialisations towards flower feeding in beetles may therefore be more prominent than originally thought, suggesting that flower feeding and associated adaptations may be an important and a relatively unexplored driving force in explaining patterns of anthophilous beetle diversity.

South African hopliine monkey beetles represent a highly speciose and promising group of flower-visiting insects that provide the opportunity for a comparative study of the morphological adaptations required to obtain various floral rewards. Their global centre of diversity and adaptive radiation is centred within South Africa with roughly 63% of the world’s species and 38% of the genera concentrated here (Colville, 2009; Holger Dombrow, 2015 provided unpublished data on global patterns of hopliine diversity to J.F.C). An impressively high percentage of species (ca. 95%) and genera (ca. 80%) are national endemics, with many genera (ca. 75%) having the majority of their species occurring within South Africa’s floristic hotspot of the Greater Cape Floristic Region (GCFR) (Colville et al., 2014).

Hopliines are frequent flower visitors and their importance as pollinators in the GCFR has been well established (Picker & Midgley, 1996; Goldblatt, Manning & Bernhardt, 1998; Goldblatt & Manning, 2011). Within this floristic region, they play a vital role in the pollination of numerous species across various plant families, including Asteraceae, Aizoaceae, Iridaceae, Proteaceae and Orchidaceae (Goldblatt & Manning, 1996; Steiner, 1998a; Steiner, 1998b; Bernhardt, 2000; Mayer, Soka & Picker, 2006). GCFR hopliines utilize flowers both as a primary food source and as mating sites (Picker & Midgley, 1996). Three pollinator guilds have been defined based on flower colour preferences, overall appearance and feeding behaviour (Picker & Midgley, 1996). This would suggest that their mouthparts are adapted towards different floristic resources, as feeding on floral tissues, pollen and nectar would require different mouthpart adaptations.

Coupled with the different feeding guilds are contrasting mating behaviours. For example, beetles perceived to be feeding for long periods on floral tissue deeply embedded inside disk-shaped daisy flowers show extreme degrees of sexual dimorphism in male-female colour and in the enlarged size of male hind legs (Picker & Midgley, 1996; Colville, 2009). Within this guild, males are highly combative, engaging in aggressive male-male contests for females and in prolonged periods of mate guarding post-copulation. In contrast, non-embedding species show lower degrees of sexual dimorphism, less combative male-male contests, and appear to feed for short periods superficially on the surface of disk-shaped and non-disk-shaped flowers on pollen and/or nectar. As such, a better understanding of mouthpart adaptations should allow insights into the dynamics between feeding adaptations and mating behaviour and observed flower visiting strategies. Therefore, understanding feeding behaviour and associated mouthpart adaptations appears essential to unlocking the drivers of this unique diversity (Stuckenberg, 1998; Karolyi et al., 2012). Furthermore, hopliines are a model group to understand why some pollinating insect groups show global centres of diversity and adaptive radiation in the GCFR (Colville et al., 2014) and how the exceptional diversity of the floristic niches found here may have promoted insect diversity (Procheş et al., 2009). In addition, hopliines also appear to be an important driver in Cape plant speciation, as seen across several plant families that show flower shape and colour adaptations specifically towards monkey beetle pollination (Steiner, 1998b; Manning et al., 2015).

The aim of the present study is therefore to compare the mouthpart morphology and content of the alimentary tract in various genera and species of South African Hopliines to reveal putative adaptations to feeding on flower tissue, pollen and nectar. The comparative examination of the micromorphology of the feeding organs and the content of the alimentary tract should enable some general insights on the feeding preferences and strategies of the different South African hopliine flower-feeding guilds. Further, results should allow for comparisons of adaptations and host plant selection, as well as feeding and mating behaviours.

## Materials and Methods

A sample of 13 out of the approximately 51 South African hopliines genera (Pèringuey, 1902) were used in this study. The selected 18 species represented all the feeding guilds as defined by Picker & Midgley (1996), as well as a non-flower feeding guild not discussed by these authors. Our range of genera sampled appears to incorporate a relatively phylogenetically diverse group (Ahrens, Scott & Vogler, 2011). All beetle species were collected on natural food plants between August and September (2010–2011) from various locations in the Northern and Western Cape Province, South Africa (Table 1). Collecting permits were provided by Northern Cape Nature Conservation (471/2010 and 472/2010) and Cape Nature (AAA004-00530-0035). Male and female individuals of all investigated species were collected and specimens were fixed in FAA solution (35% formalin, 100% glacial acetic acid, 90% alcohol) and later preserved in 70% ethanol.

**Table 1:** Hopliine species, sampling sites and gut content. Sites are Western Cape: BP Botterkloof Pass (31°51′43.45″ S,19°16′14.26″E), CP Cape Peninsula (34°05′57.57″S, 18°21′37.22″E), DL Darling (33°23′55.41″S, 18°24′07.19″E) and MB Malmesbury (33°26′11.57″S, 18°32′26.06″E); Northern Cape: NGR Nieuwoudtville Grasberg Road (31°20′54″S, 19°05′30″E), NHBG Nieuwoudtville Hantam Botanical Garden (31°24′43″S, 19°09′43″E) and KK Kamieskroon (30°44′11″S, 18°6′57″E).

Species	Sampling site	Gut content
*Anisochelus inornatus* Burmeister, 1844	NGR, NHBG	floral tissue
*Anisonyx ursus* Fabricius, 1775	DL	nectar
*Chasme decora* Wiedemann, 1823	MB	pollen
*Chasme* sp.	KK	floral tissue
*Clania glenlyonensis* Dombrow, 1997	NGR, NHBG	floral tissue
*Clania macgregori* Dombrow, 1997	NGR, NHBG	empty
*Congella* sp.	CP	foliar tissue
*Dolichiomicroscelis gracilis* Pèringuey, 1902	DL	floral tissue
*Heterochelus pickeri* Dombrow, 1997	NHBG	empty
*Kubousa gentilis* Pèringuey, 1902	MB	floral tissue
*Lepisia ornatissima* Burmeister, 1844	KK	floral tissue
*Lepisia rupicola* Fabricius, 1775	DL	floral tissue
*Lepithrix* sp.	KK	pollen
*Mauromecistoplia nieuwoudtvillensis* Dombrow, 1997	BP	floral tissue
*Pachycnema calcarata* Burmeister, 1844	KK	nectar
*Pachycnema crassipes* Fabricius, 1775	NHBG, NGR	pollen
*Pachycnema flavolineata* Burmeister, 1844	KK	pollen
*Scelophysa scheffoldi* Dombrow, 1999	KK	floral tissue

### Light microscope

Heads were removed from the body and imbedded in a melted wax-rosin mixture with a soldering iron and insect pins. Mouthparts were dissected using a stereo microscope and embedded in Polyvinyllactophenol on a microscopic slide. After drying for 48 hours specimen were sealed with nail polish to prevent them from draining and to avoid infiltration of air. Images of different focal planes were taken with an Olympus CX41 light microscope equipped with a digital Olympus E330 camera. Micrographs were stitched using Helicon Focus software (Version 3.10) and processed with Adobe Photoshop CS5 (Version 12.0). For each of the 18 collected species, between 3 to 11 specimens were investigated in detail.

The contents of the anterior gut were extracted using a stereo microscope and microscope slides were prepared in Polyvinyllactophenol. After air drying, the gut contents were examined using a light microscope (Olympus CX41).

### Scanning Electron Microscope (SEM)

Heads and dissected mouthparts were dehydrated in absolute ethanol and submerged in Hexamethyldisilazan. After air drying overnight, specimens were attached to an aluminium stub with graphite adhesive tape and sputter-coated with gold (240 s, Agar sputtercoater B3740). Micrographs were taken from 1 to 3 specimens of selected representatives with a Philips XL 20 SEM and a Philips XL 30 ESEM (Philips, Amsterdam, Netherlands).

### Statistical analysis

Data analyses were conducted using Correspondence Analysis (CA) with the statistical computing software R 3.2.2 (R Core Team, 2015). This analysis allows trait and feeding guild to be statistically related. Results were visualized with a balloon plot (function balloonplot) in the “gplots” package (Warnes et al., 2015), which uses a contingency table based on a character matrix of mouthpart traits and graphically shows the relative magnitude of these traits to different feeding guilds. The contingency table was built using the package “factoMineR” (Husson et al., 2015) and the function “devtools” within the “factoextra” package (Kassambara, 2015).

## Results

### General mouthpart morphology

All investigated species displayed prognathous mouthparts that consisted of the labrum with the epipharynx, paired mandibles and maxillae, as well as the labium with the hypopharynx. The maxillary and labial palps were found to be similar in size and shape across all studied species. Mouthparts were found to differ only in size, but not in form between sexes. Consequently, no conclusions about differences in the feeding preferences between males and females were drawn.

#### Labrum and epipharynx

**Figure 1:**
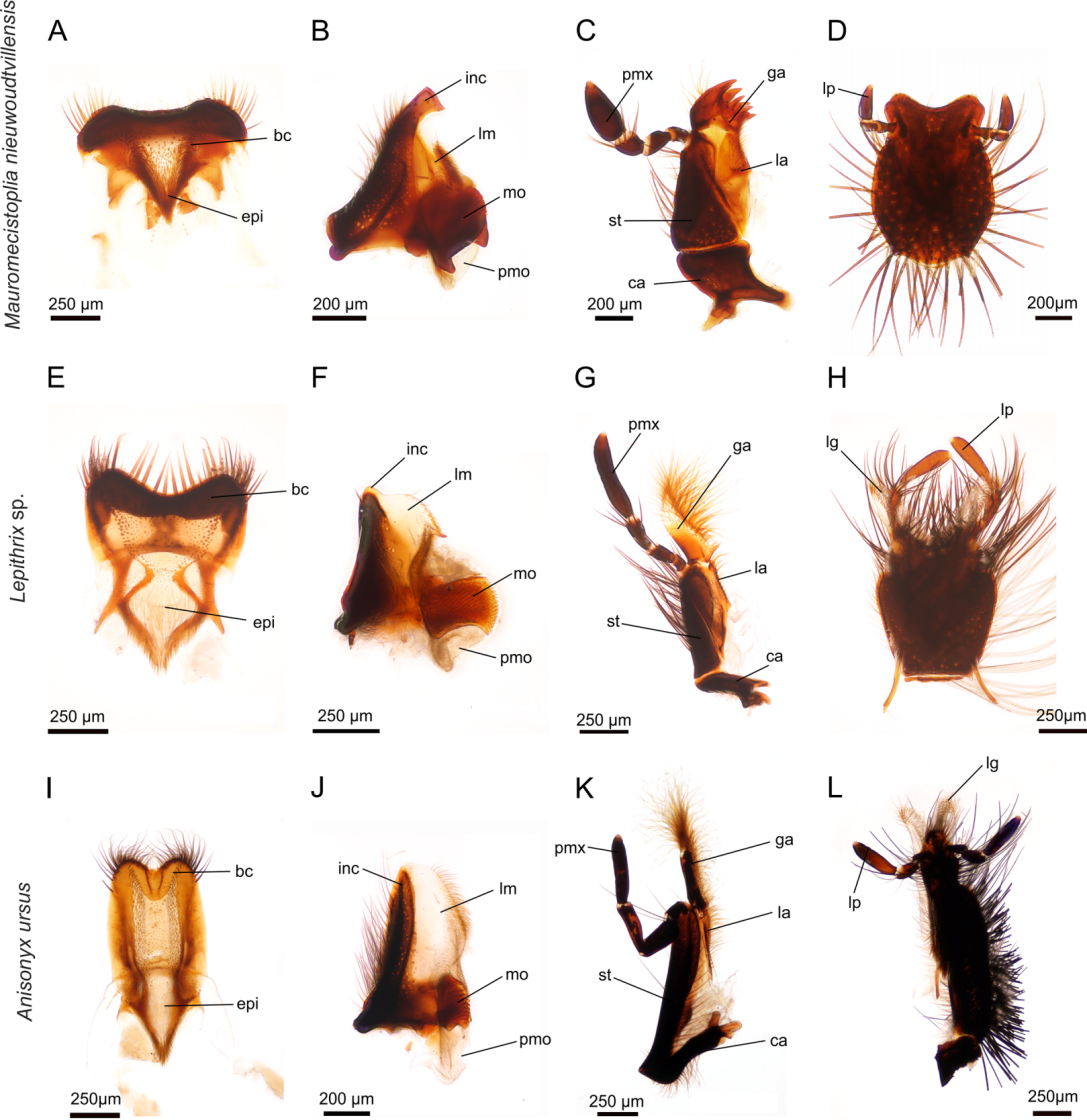
Mouthparts of three different hopliine species according to their designated feeding guilds. (A–D) heavy sclerotized mouthparts of flower-feeding *Mauromecistoplia nieuwoudtvillensis*: (A) labrum with hair crest and epipharynx. (B) mandible with conspicuously formed incisive and prominent molar part. (C) maxilla with sclerotized teeth on the galea. (D) rounded labium. (E–H) mouthparts of pollen-feeding *Lepithrix* sp. (E) labrum with hair crest and epipharynx. (F) mandible displays a membranous lacinia mobilis with a proximal, sclerotized tooth and well-developed molar region. (G) maxilla with haired, membranous galea. (H) labium. (I–L) elongated mouthparts of nectar-feeding *Anisonyx ursus*. (I) weaker sclerotized labrum and epipharynx (J) mandible with prominent, membranous lacinia mobilis and relatively small molar part. (K) maxilla with conspicuously haired galea. (L) elongated labium. bc, hair crest; epi, epipharynx; ca, cardo; ga, galea; inc, incisivus; la, lacinia; lg, ligula; lm, lacinia mobilis; lp, labial palpus; mo, mola; pm, postmola; pmx, palpus maxillaris; st stipes.

**Figure 2:**
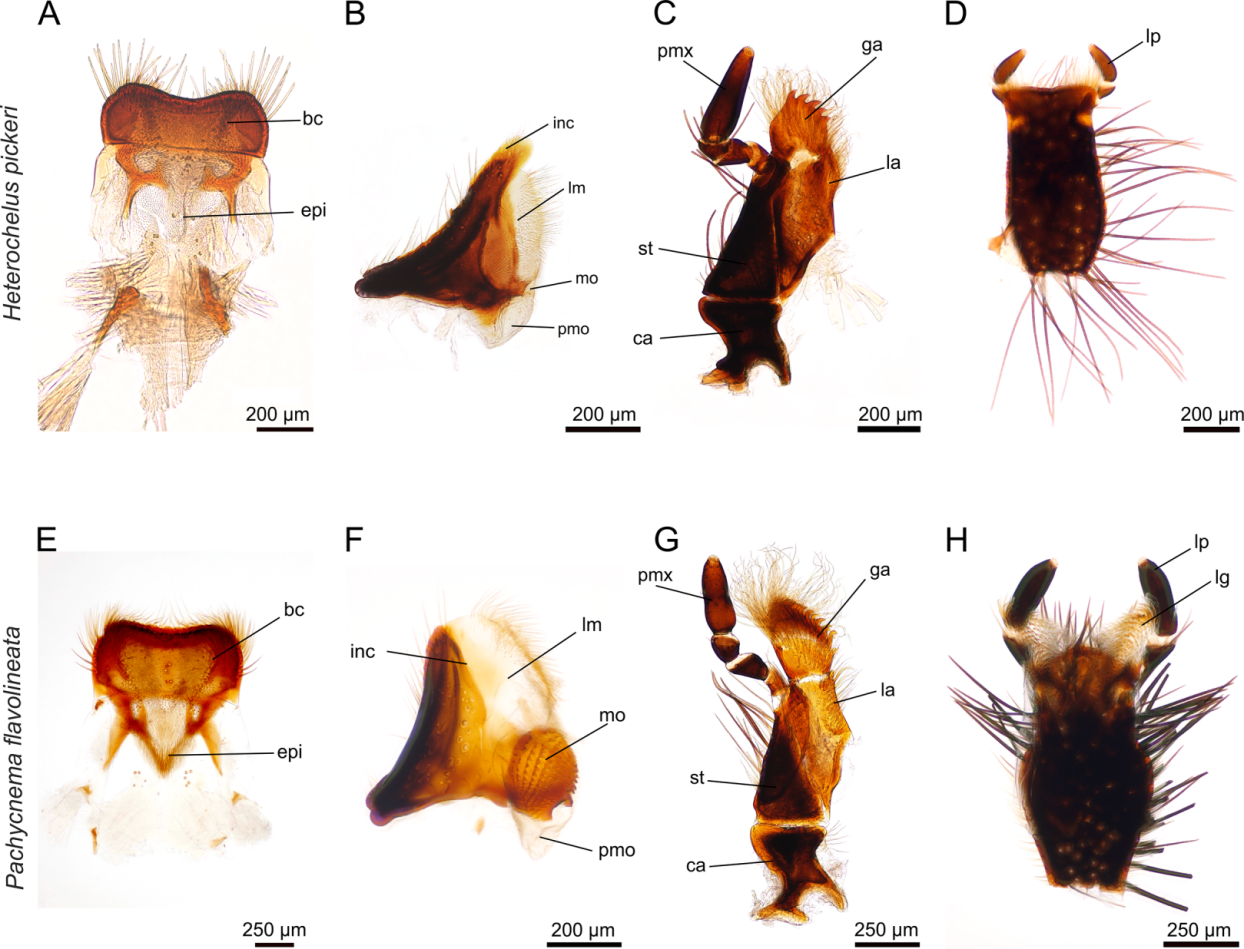
Mouthparts of *Heterochelus pickeri* (A–D) and *Pachycnema flavolineata* (E–H). (A) labrum with hair crest and epipharynx. (B) mandible displays a particularly haired lacinia mobilis but the molar part is reduced. (C) maxillae with haired galea and lacinia; furthermore, the galea is conspicuously toothed. (D) ventral view of the labium. (E) labrum with hair crest and epipharynx. (F) mandible with large, membranous and haired lacinia mobilis and prominent molar part. (G) maxilla with long, curly hair on galea and lacinia; the galea additionally carries rows of hooked teeth. (H) ventral view of the labium with elongated, membranous ligulae. bc, hair crest; epi, epipharynx; ca, cardo; ga, galea; inc, incisivus; la, lacinia; lg, ligula; lm, lacinia mobilis; lp, labial palpus; mo, mola; pm, postmola; pmo, palpus maxillaris; st, stipes.

The labrum, together with the clypeus, formed the cibarial roof. Overall, the labrum of all investigated species was a sclerotized, more or less heart-shaped structure, equipped with long hairs, particularly on the concave distal edge (Figs. 1A, 1E, 1I, 2A and 2E). The latter also showed strong sclerotization with lateral bulges, and ventrally a v-shaped hair crest composed of different numbers of setae was seen connected to the membranous, haired epipharynx. In contrast to the other investigated species, the labrum of *Anisonyx ursus*, *Pachycnema grassipens* and *P. calcarata* was weakly sclerotized. Further, in the case of *A. ursus*, it was elongated and slender in appearance with a u-shaped indentation on the distal edge (Fig. 1I).

#### Mandibles

**Figure 3:**
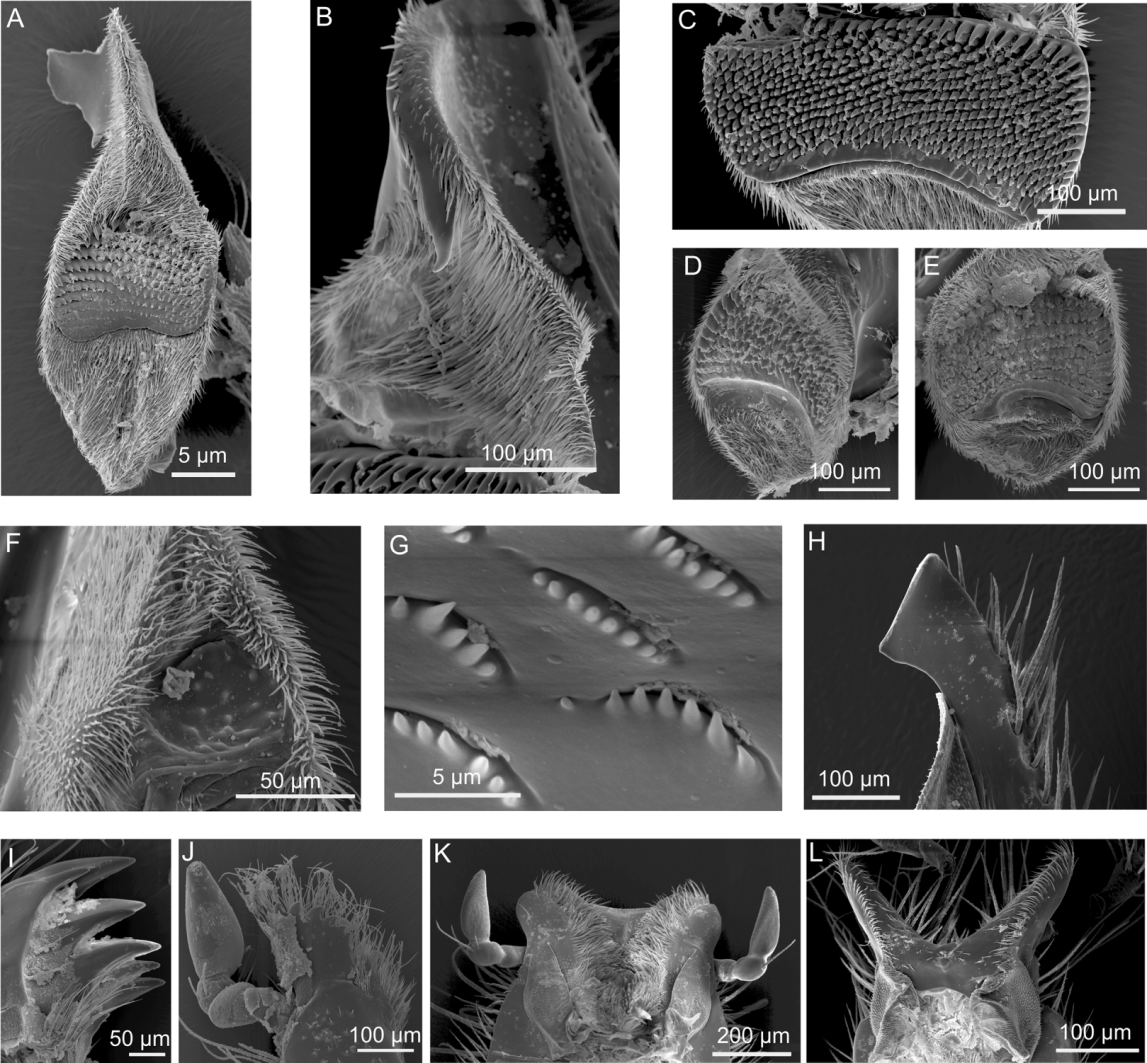
SEM micrographs of the mouthpart of various hopliine species. (A) *Anisonyx ursus*, median view of the mandible with lacinia mobilis, molar surface and haired postmola. (B, C) *Lepithrix* sp., lateral view of the mandible with single tooth on lacinia mobilis and toothed mola surface. (D, E) *Pachycnema flavolineata*, mola of the left and right mandible. (F) *Heterochelus pickeri*, reduced molar part of the mandible. (G–I) *Mauromecistoplia nieuwoudtvillensis*. (G) rows of teeth on the lacinia mobilis, (H) incisivus with cutting edge and (I) toothed galea. (J) *Heterochelus pickeri*, setose, toothed galea. (K) dorsal view of the labium with hypopharynx of *M. nieuwoudtvillensis* and (L) *Lepithrix* sp. showing apical elongated ligulae.

The paired, stout mandibles across all the hopliine species examined were mostly rectangular in shape. Each mandible consisted of a proximal, heavily sclerotized mola with a membranous pilose postmola, a membranous lacinia mobilis and a distal blade-like incisivus (Figs. 1B, 1F, 1J, 2B, 2F and 3A–3H). The strongly sclerotized mola was a socket-shaped structure with a grinding surface equipped with several rows of small pointed teeth. In contrast to all other investigated species, the left and right mola of *Pachycnema flavolineata* differed concerning the surface structure. On the surface of the right mola, a sclerotized, curved ledge could be found; the left molar displayed a corresponding ridge (Figs. 3D and 3E). *Heterochelus pickeri*, contrasted strongly with all other species in that it showed a significantly reduced molar (Fig. 3F), resulting in a slim, triangular shaped mandible (Fig. 2B).

The main part of the mandible for all hopliine species was formed by the convex lacinia mobilis and this more or less pilous lobe reached from the mola to the incisivus. In some species (e.g. *Mauromecistoplia nieuwoudtvillensis*) the lacinia mobilis was covered with rows of short, tooth-shaped structures (Fig. 3G). *Clania* species, *Congella* species, *Lepisia* species and *Scelophysa scheffoldi* possessed a single, lateral tooth on the lacinia mobilis. In *Chasme decora* and *Lepithrix* sp. only a few hairs were found on the lacinia mobilis, but a conspicuously hooked tooth protruded at the base (Figs. 1F and 3B). In *A. ursus*, and in all three *Pachycnema* species, the mandible was equipped with a conspicuously large, square-shaped and soft lacinia mobilis (Figs. 1J and 2F).

Across most species the smooth, rounded incisivus was sclerotized, usually lacked a cutting edge and protruded distally from the heavily sclerotized basal joint region. In *Congella* sp., *Kobousa gentilis* and *M. nieuwoudtvillensis* a cutting edge was, however, evident, formed by the bent apex of the incisivus (Figs. 1B and 3H).

#### Maxillae

The paired maxillae generally consisted of the basal cardo with forked apodeme, the stipes with a lacinia and a well-developed galea distally. Laterally, the four-segmented maxillary palp was attached to the distal region of the stipes and reached different lengths (Figs. 1C, 1G, 1K, 2C and 2G). The cardo appeared mostly short and stout, but in *A. ursus*, *P. calcarata* and *P. crassipes* the maxillae were particularly elongated and slender.

Across most species, the lacinia appeared as a small, sclerotized lobe (e.g. *H. pickeri*, Fig. 2C) or ledge (e.g. *Lepithrix* sp. and *A. ursus*, Figs. 1G and 1H) with individual short setae. Only *M. nieuwoudtvillensis* and *P. flavolineata* showed a well-developed lacinia (Figs. 1C and 2G). In contrast, the galea was usually equipped with numerous long, mostly curled hairs. The galea of *A. ursus*, *P. calcarata* and *P. crassipes* was particularly elongated, with densely haired lateral and apical regions. The galea of *A. ursus* was also noticeably developed as a long, slender and heavily sclerotized sclerite. Further, *C. decora* and *Lepithrix* sp. possessed a sclerotized, oblique galea showing only a few thick hairs apically (Fig. 1G). In most of the investigated species, the galea additionally bore a few large, sclerotized and pointy teeth (e.g. *M. nieuwoudtvillensis*, Figs. 1C and 3I). Furthermore, *H. pickeri* and *P. flavolineata* possessed a continuous row of saw-like teeth in combination with long curled hairs (Figs. 2C, 2G and 3J).

#### Labium

Across all species, the strongly sclerotized, broad labium formed the floor of the cibarium and was covered ventrally with numerous, long hairs. On the dorsal side, a v-shaped crest of hairs, consisting of densely packed setae, was connected to the hypopharynx (Figs. 1D, 1H, 1L, 2D, 2H and 3K). The paired ligulae appeared as membranous, setose lobes on the distal margin and formed a forked extension of the prementum, situated between the three-segmented labial palps (Fig. 3L). Both ligulae and labial palps were found to be of varying lengths. A row of setae was situated on the dorsal edges of the otherwise smooth ligula in all species. In some species (e.g. *H. pickeri*), the ligulae were found to be reduced (Figs. 1D and 2D), or consisted of short, lateral lobes on the distal margin (e.g. *M. nieuwoudtvillensis*, Fig. 1D). In *A. ursus*, *P. calcarata* and *P. crassipes* the labium was noticeable longer and more slender and the elongated, membranous ligulae were nearly as long as the labial palps (Fig. 1L).

### Content of the alimentary tract

Flower tissue was found in the guts of both *Clania* species, *Chasme* sp., *Congella* sp., *Dolichiomicroscelis gracilis*, *Kubousa gentilis*, both *Lepisia* species, *Scelophysa scheffoldi* and *M. nieuwoudtvillensis*. In addition, various amounts of intact pollen grains could be found on the mouthparts and in the guts of each of these species. All dissected specimens of *C. decora*, *P. crassipes* and *P. flavolineata* had large amounts of intact pollen grains in their guts, with some grains found on the mouthparts as well. No pollen grains could be found in the gut of *A. ursus* and *P. calcarata*; although a colourless or orange substance was found in the foreguts of both species. While no pollen grains were found in the dissected gut of *H. pickeri*, small amounts were found on the mouthparts.

### Correspondence between characters and feeding guilds

**Figure 4:**
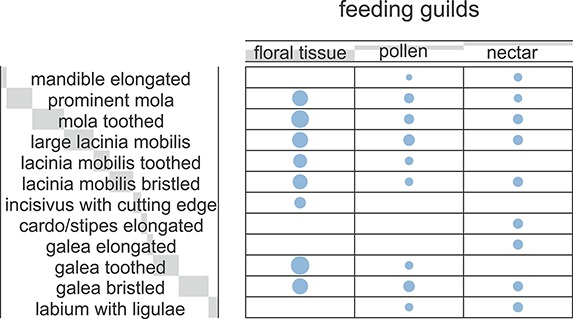
Ballon plot of hopliine feeding guilds. Blue circles represent the relative magnitude of the corresponding mouthpart characters. The size of the blue circles proportionally reflects the frequency of data for each character and the associated feeding guilds. Light grey bars behind headers indicate row and column sums, with bar lengths being proportional to the corresponding sum.

**Figure 5:**
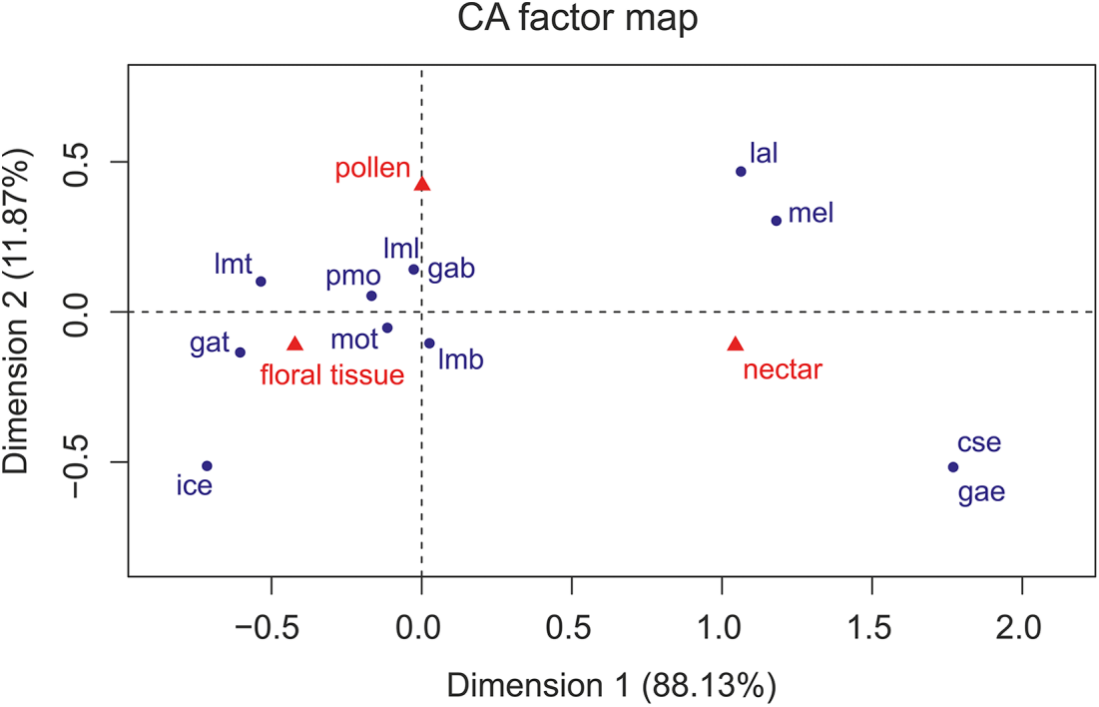
Correspondence analysis factor map. Mouthpart characters are represented by blue points and feeding guilds by red triangles. The distances between characters or feeding guilds give a measure of their similarity. mel, mandible elongated; pmo, prominent mola; mot, mola toothed; lml, lacinia mobilis large; lmt, lacinia mobilis toothed; lmb, lacinia mobilis bristled; ice, incisivus with cutting edge; cse, cardo/stipes elongated; gae, galea elongated; gat, galea toothed; gab, galea bristled; lal, labium with ligulae.

The Correspondence Analyses (CA) indicated a significant association between mouthpart characters and feeding guilds (*r* = 0.63; Chi-square of independence, χ^2^(22) = 43.48, p = 0.004). The balloon plot (Fig. 4) displays the relative magnitude of characters to different feeding guilds of the investigated species, and the CA factor map (Fig. 5) gives a measure of similarity between mouthpart characters (blue points) and feeding guilds (red triangles). Elongated mouthpart structures (mel, cse and gae) and ligulae of the labium (lal) corresponded with nectar feeders. In addition, pollen and floral tissue feeders corresponded closely with a prominent, toothed mola (pmo, mot) and a large, densely bristled (lml, lmb) and sometimes toothed lacinia mobilis (lmt) as well as a bristled galea (gab). Incisivi with cutting edges (ice) and toothed galeae (gat) were only found in the floral tissue feeding guild. This indicated that, although pollen- and floral-tissue feeders shared some characters, the three feeding guilds could be distinguished from each other by reference to their mouthpart morphology.

## Discussion

Hopliines form a diverse group of flower-visiting beetles with an almost worldwide distribution, although their greatest diversity occurs in the Greater Cape Floristic Region of South Africa where they appear to have coevolved with the diverse endemic flora (Colville, 2009; Goldblatt & Manning, 2011). Based on mostly behavioural observations, they have been purported to feed on various floral food sources and have been observed to visit flowers for additional reasons, including for shelter and as mating sites (Johnson, 2004; Nicolson, 2007). The present study revealed different mouthpart adaptations in South African hopliines specialised towards different food preferences. However, we noted some contrasting findings between mouthpart morphology and guild structure, as defined by Picker & Midgley (1996), across different genera and species. We suggest that these mismatches between mouthpart morphology and flower visiting behaviours requires alternative explanations, not necessarily associated with feeding preferences.

### General hopliine mouthpart structure

Both the labrum and labium, with the haired epipharynx and hypopharynx, respectively, serve as the dorsal and ventral cover of the cibarium and seal the oral cavity. While the maxillae represent the primary nutritional uptake organs in scarab beetles, the mandibles are responsible for the processing of food within the cibarium (Karolyi, Gorb & Krenn, 2009). In hopliines, the labrum and labium showed fewer modifications among the feeding groups than the maxilla and the mandible.

Pèringuey (1902) characterized the maxilla of hopliines as bilobated (inner lobe, the lacinia; and outer lobe, the galea), whereby the lacinia is often indistinct or absent and is always immovable (see also Williams, 1938). These first descriptions of the maxilla coincide broadly with those found in the present study. The inner lobe, the lacinia, which usually represents the sclerotized and robust part of the maxilla in scarab beetles, was reduced in hopliines. In the hopliine feeding groups that target nectar and pollen, the lacinia, although reduced, was found to be covered with pads of hairs. These are most likely adaptations towards mopping-up nectar and sweeping-up pollen. The elongated, basal cardo possibly allows an increased movability in protruding the maxilla to gather pollen and transport it to the mouth (Schremmer, 1961; Fuchs, 1974; Johnson & Nicolson, 2001; Krenn, Plant & Szucsich, 2005). As such, we here show that hopliines show several modifications of the maxilla towards specialised and different feeding strategies.

### Classification of hopliine feeding groups

Based on their mouthpart morphology and gut content, the investigated species were classified into three different feeding groups: floral/plant tissue feeders; pollen and nectar feeders (Table 2; Figs. 1 and 2) and one species that we could not place accurately, but which may have an intermediate feeding strategy utilizing floral tissue, pollen and perhaps nectar (Fig. 2). Although the gut content analyses helped guide the understanding of mouthpart specialisations towards feeding on a particular floral resource, greater sampling of individuals would be required to confirm this. Our results were mostly based on a few random specimens per species. Future studies should more systematically sample individuals from all host plant species being used. Additionally, sampling of individuals of a species from different plant communities and habitats would confirm if a particular floral resource is targeted irrespective of the changing plant species in a community. As such, our gut content analyses should be treated with some caution and considered as preliminary at this stage.

**Table 2:** Characteristic features of the mouthparts in the feeding guilds of specialised, flower visiting Hopliines in alphabetical order.

Species	Labrum	Mandibles			Maxillae			Labium		Feeding group	Feeding guild^a^
		Mola	Lacinia mobilis	Incisivus	Cardo/stipes	Lacinia	Galea	Form	Ligula		
*Anisochelus inornatus*	broad, sclerotized	toothed	small lobe	rounded	stout	developed	toothed setose	broad	reduced	floral tissue	pollen/nectar
*Anisonyx ursus*	elongated, weak sclerotized	small, toothed	large	rounded	long, slender	small ledge	elongated, sclerotized brush	elongated	elongated	nectar	pollen/nectar
*Chasme decora*	broad, sclerotized	toothed	medium large	rounded	stout	small ledge, few setae	brush-like	broad	reduced	pollen	pollen/nectar
*Chasme sp.*	broad, sclerotized	toothed	medium large, toothed	rounded	stout	developed	toothed setose	broad	reduced	floral tissue	pollen/nectar
*Clania glenlyonensis*	broad, sclerotized	toothed	medium lobe, toothed	rounded	stout	small ledge, few setae	brush-like, toothed	broad	reduced	floral tissue	pollen/nectar
*Clania macgregori*	broad, sclerotized	toothed	medium lobe, toothed	rounded	stout	developed	toothed, setose	broad	reduced	floral tissue	pollen/nectar
*Congella sp.*	broad, sclerotized	toothed	medium lobe	hooked cutting edge	stout	developed	toothed	broad	reduced	foliage tissue	NA
*Dolichiomicroscelis gracilis*	broad, sclerotized	large, toothed	medium lobe, toothed	broad, rounded	stout	small ledge, few setae	toothed, setose	broad	small	floral tissue	pollen/nectar
*Heterochelus pickeri*	broad, sclerotized	reduced	long, setose lobe	rounded	stout	small, setose lobe	brush-like, row of teeth	rectangular, elongated	reduced	pollen?	floral tissue/pollen
*Kubousa gentilis*	broad, sclerotized	small, toothed	small lobe	hooked cutting edge	stout	small ledge	toothed, setose	broad	reduced	floral tissue	pollen/nectar
*Lepisia ornatissima*	broad, sclerotized	large, toothed	medium lobe, toothed	rounded	stout	developed	brush-like, toothed	broad	reduced	floral tissue	pollen/nectar
*Lepisia rupicola*	broad, sclerotized	large, toothed	medium lobe, toothed	rounded	stout	small ledge	toothed, setose	broad	reduced	floral tissue	floral tissue/pollen
*Lepithrix sp.*	broad, sclerotized	large, toothed	medium lobe, with hooked tooth	rounded	slender	small ledge	brush-like	broad	elongated	pollen	pollen
*Mauromecistoplia nieuwoudtvillensis*	small, broad, sclerotized	large, toothed	small lobe, toothed	hooked cutting edge	stout	developed	toothed, few setae	broad	reduced	floral tissue	floral tissue/pollen
*Pachycnema calcarata*	broad, weak sclerotized	small, toothed	large, setose lobe	rounded	slender	small ledge	elongated, brush-like	elongated	elongated	nectar	floral tissue/pollen
*Pachycnema crassipes*	broad, weak sclerotized	large, toothed	long, setose lobe	rounded	long, slender	developed	elongated, brush-like	elongated	elongated	nectar	floral tissue/pollen
*Pachycnema flavolineata*	broad, short	large, toothed	large, setose lobe	rounded	stout	setose ledge	brush-like, row of teeth	broad	elongated	pollen	floral tissue/pollen
*Scelophysa scheffoldi*	broad, short, sclerotized	large, toothed	medium lobe, toothed	rounded	stout	developed	toothed, few setae	broad	reduced	floral tissue	floral tissue/pollen

Note:

a Picker & Midgley (1996).

### Feeding on floral and foliage tissues

Ten out of the 18 investigated hopliine species were classified as feeding on flower tissue, and one species on foliage tissue (Table 2). Hopliines characterised as floral or foliage tissue feeders displayed a similar mouthpart morphology characterized by teeth and cutting edges on the maxillae and mandibles. Picker & Midgley (1996; see also Midgley, 1993) regarded such species as being destructive to their host flowers. Several differences in the morphology of the mandibles, especially in the shape of the incisivus, were found in this group and not in others. The sclerotized cutting edges on the mandibles, together with the toothed galea, are considered as adaptations towards floral tissue feeding. The rows of short, tooth-shaped hairs as well as the single, lateral tooth found in some species (e.g. *Clania*) on the lacinia mobilis of the mandible are possibly used to grate floral or foliage tissue, whereas the sclerotized molar is most likely used to grind floral particles. Hopliines characterized as flower and foliage feeders were found to be the only ones with cellulose present in their gut; although not all specimens in this group contained cellulose in their guts.

### Pollen-feeding

The challenges confronting insect pollen feeders are the handling and removal of pollen grains and gaining access to the nutrients encapsulated in the hard and highly resistant exine. Pollen is a valuable food source for flower visiting insects since it contains various lipids, carbohydrates and proteins (Roulston & Cane, 2000; Johnson & Nicolson, 2001; Cook, Murray & Williams, 2004). Characteristic adaptations of pollen feeding in Coleoptera include elongated maxillae with variously shaped combs and hairs that function as pollen harvesting devices (Fuchs, 1974). In Coleoptera, the mandibles are particularly adapted for pollen manipulation and consist largely of the lacinia mobilis (or prostheca), a soft and usually bristly lobe, and the postmola, which together knead the pollen (Schremmer, 1961; Fuchs, 1974; Nel & Scholtz, 1990). The pollen grains are conveyed into the actual mouth opening by coordinated movements of the mouthparts (Fuchs, 1974; Karolyi, Gorb & Krenn, 2009).

Only three species of hopliine (Table 2) based on the general mouthpart characteristics of pollen feeders as described above, could be classified as specialised pollen-feeders. Adaptations for pollen-feeding in these species can be seen in several modifications of the mandible and the maxilla. The lobe-like lacinia mobilis, with its hooked tooth, most likely functions as a pollen collecting device inside the cibarium, as seen in other scarab beetles, where during movement of the mandibles, the lacinia mobilis acts as a scraper, wiping pollen grains to the molar part. The two hopliine species in this specialised pollen-feeding group possessed a prominent mola with numerous rows of teeth that may be used for perforating pollen grains.

In pollen-feeding Scarabaeidae, the distal part of the maxilla, i.e., the haired galea, is considered as the primary organ for pollen uptake, and the sclerotized hairs are expected to function as an ideal pollen brush (Karolyi, Gorb & Krenn, 2009). Although the lacinia was found to be reduced in these specialised hopliine pollen-feeders, the hair comb of the lacinia most likely is used to convey pollen grains to the mandibles. In nectar- and pollen-feeding Meloidae, the haired ligulae are considered specialised organs for harvesting pollen and nectar (Wilhelmi & Krenn, 2012). Therefore, the forward pointing ligulae on the labium of pollen-feeding hopliines are hypothesised to have the same function.

Since most of the pollen grains found in the gut of pollen-feeding species were intact, it can be suggested that the broad molae of the mandibles are most likely not used to crack open pollen. South African protea beetles (Cetoniinae) and some monkey beetles are known to use osmotic damaging of the pollen wall inside the gut, enabling digestive enzymes to enter via the pores (Johnson & Nicolson, 2001; see Karolyi, Gorb & Krenn, 2009 for summary of various mechanisms of pollen digestion in Coleoptera). The teeth found on the molar of investigated species might therefore be used primarily to pierce pollen grains, enabling digestive enzymes to enter within the gut.

### Nectar-feeding

Sugar concentrations in nectar mostly range between 15–60%, depending on the plant species (Daniel, Kingsolver & Meyhöfer, 1989). In addition, nectar contains small amounts of amino acids, proteins, organic acids, phosphates, vitamins and enzymes (Baker & Baker, 1973; Kevan & Baker, 1983; Nicolson, 2007). Sugars in nectar provide the primary energy source for fast flying flower visitors that have high energetic requirements, such as hummingbirds and various insects. Although, several south African Iridaceae offer nectar to insects with elongated mouthparts, like long-proboscid Diptera (Goldblatt & Manning, 2000; Johnson, 2004; Karolyi et al., 2012; Karolyi et al., 2013; Karolyi et al., 2014), some plant species have adapted their flower morphology towards attracting hopliines with secreted nectar (Goldblatt & Manning, 2011). Picker & Midgley (1996) classed species of *Peritrichia* and *Anisonyx* as nectar feeders. However, no species of *Peritrichia* examined in this study were determined to be nectar feeders; only *A. ursus* and *Pachycnema calcarata* and *P. crassipes* were identified as specialised nectar feeders (Table 2). Picker & Midgley (1996) classed species of *Pachycnema* as pollen and floral tissue feeders based on their general behaviour of embedding deeply within the capitulum of flowers.

The three investigated species characterised as nectar feeders possessed long and slender mouthparts that lacked any cutting edges that could be used for damaging their host plants (Figs. 1I–1L). The labrum differed in shape and was rather weakly sclerotized compared to the flower and pollen-feeding species. Furthermore, the broad shape and soft texture of the lacinia mobilis suggests the ability to sweep nectar into the mouth within the cibarium. The long and slender maxillae, with the hirsute and heavy sclerotized galea, are most likely adapted for probing into flowers and for mopping-up nectar. The long and slender cardo found in nectar feeding hopliine species presumably allows protrusion and a higher movability of the distal maxilla parts during food uptake. In addition, the elongated labium, together with the elongated, hirsute ligulae, was also considered to be an adaptation towards nectar feeding in these three hopliine species.

### Florivory, pollen or nectar feeding, or a mixed cocktail?

Some of the investigated species showed mouthpart adaptations for a dual diet, suggesting that these species may feed on more than one type of floral food source. In particular, *Heterochelus pickeri* and *Pachycnema flavolineata* could not clearly be categorized into a specific feeding group because their mouthparts displayed adaptations to several different food sources. Although the maxillae appeared quite similar between the two species, the mandibles differed significantly.

Picker & Midgley (1996) described *Heterochelus* species as being destructive to their host plant´s flowers through their feeding behaviour. Concerning the mandibles of *H. pickeri*, this seems contradictory since they possessed no cutting edges and the reduced mola was unsuitable to grind or crush any food particles. Further, the lacinia mobilis, together with the postmola, appear to function rather as a conveying structure for pollen and/or nectar, like in European *Cetonia* beetles (Karolyi, Gorb & Krenn, 2009). In addition, the galea brushes most likely serve as effective pollen up-taking organs. From its position in the head capsule, the toothed galea seemed better suited towards combing pollen grains, rather than for feeding on floral tissue. Compared to the floral tissue feeding species, the galea teeth of both *H. pickeri* and *P. flavolineata* were rather short, possibly functioning more as a pollen rasp, grating pollen from the anthers. Although the mandible of *H. pickeri* appears not to be adapted to grind pollen, beetles may ingest pollen intact and digest these via an osmotic gradient in the gut, as described for other hopliines (Johnson & Nicolson, 2001).

In *P. flavolineata*, the shape of the mandible suggests that this species feeds on pollen. Modifications of the mandible, such as a large, hairy lacinia mobilis and postmola, as well as a strongly sclerotized mola, have been reported for beetles feeding on pollen and nectar (Krenn, Plant & Szucsich, 2005). In addition, the surface of the mola might function like a tongue and groove joint, interlocking the mandibles during feeding. This might enhance the force that can be applied to crush pollen grains. The combination of the toothed, but also hairy galea of the maxillae of *P. flavolineata*, together with the well-developed ligulae of the labium represents a combination of traits for pollen uptake as well as floral tissue feeding. This suggests a twofold diet for this species, although, based on the overall shape of the mouthparts, it can be concluded that pollen possibly represents the primary food source. This was confirmed by gut content analysis, where several specimens were found to have numerous Asteraceae pollen grains in the gut.

### Relating mouthpart adaptations to flower visiting behaviour

Although some matches between monkey beetle pollinator guild placements by Picker & Midgley (1996) could be confirmed with the results of this study, several of the former classifications do not conform to current findings. According to the mouthpart structure of the species examined here, species that have previously been classified as pollen and/or nectar feeders should now be considered as primarily floral tissue feeders (e.g. *Clania* species), whereas floral tissue and/or pollen feeders should now be regarded more as nectar and pollen feeders (e.g. *Pachycnema* species). Most of the feeding preferences have been derived from their flower visiting behaviour. However, the results presented here suggests that not all flower visiting behaviours are linked with feeding and selection on the primary food source.

Beetles belonging to the embedding group mostly visit Asteraceae and Aizoaceae and are typically found buried deeply into flowers (Picker & Midgley, 1996). Because of this behaviour, species of this group have been considered destructive to their host flowers and their role as pollinators have been questioned. However, according to their mouthpart morphology, only *Scelophysa scheffoldi* can be regarded as a strictly floral tissue feeder, while the other species within this group are nectar and/or pollen feeders. The embedding behaviour may therefore be due to reasons other than feeding on floral tissue, e.g. possibly related to predator avoidance (Ryan, Colville & Picker, 2013; see also Colville, 2009 for additional hypotheses associated with the embedding behaviour in hopliines). Consequently, some hopliine species can be destructive to their host flowers because of their burrowing and not necessarily their feeding behaviour.

Because of the mismatches found between pollinator guilds established by Picker & Midgley (1996) and the feeding groups described in this study, current guild definitions of hopliines need refining so that mouthpart morphology is also considered.

## Conclusions

The presented study has shown that investigated species of monkey beetles could be classified into feeding groups according to their mouthpart morphology and that monkey beetle species show adaptations towards specialised flower feeding. The differentiation between strictly pollen and nectar feeding adaptations, however, was less clear, suggesting that most species specialise on more than one floral resource. Although detailed investigations of the mouthparts allowed conclusions about feeding preference, further morphological and behavioural investigations as well as detailed gut content analyses are necessary to study hopliine host plant relationships in the field. Hopliines appear to have partitioned the flower resources in terms of feeding preferences, mating behaviour and other behavioural traits such as predator avoidance and therefore an interdisciplinary approach is required in order to better understand the flower visiting behaviour, food preferences and feeding ecology of these important pollinating beetles. The hopliines have undergone spectacular adaptive radiation in the Greater Cape Region with many highly speciose genera, e.g. *Heterochelus* with >140 species (Pèringuey, 1902). A greater sampling of this diversity across different floral habitats would be required to fully assess the role of feeding specialisations and mouthpart adaptations on the diversification of Cape hopliines. In this context, phylogenetic distributions of mouthpart adaptations are not yet known, thus the ancestral condition and evolution of feeding specialisations within Cape hopliines must remain speculative at this stage. Placing such patterns of feeding specialisations within a phylogenetic context is a necessary step in interpreting the evolution of host choice and feeding behaviour of hopliines. Without adequate phylogenetic data it is difficult to conclusively link feeding associations with ecological explanations, and to generalize trends across the tribe.

Nonetheless, together with previous studies about long-proboscid flies (Karolyi et al., 2012; Karolyi et al., 2013; Karolyi et al., 2014), this study contributes to the understanding of the functional morphology and evolution of the mouthparts of important pollinators in the Greater Cape Floristic Region. Furthermore, this study gives additional insights into the spectacular diversity of specialised pollination systems in this global floristic hotspot.

## Supplemental Information

10.7717/peerj.1597/supp-1 Supplemental Information 1 Appendix A: List of hopliines mouthparts characters.

10.7717/peerj.1597/supp-2 Supplemental Information 2 Appendix B: Character state matrix.

10.7717/peerj.1597/supp-3 Supplemental Information 3 Appendix C: Contingency table.

10.7717/peerj.1597/supp-4 Supplemental Information 4 raw data of mouthpart measurements.

## Additional Information and Declarations

### Field Study Permissions

The following information was supplied relating to field study approvals (i.e., approving body and any reference numbers): Northern Cape Nature Conservation, permits 471/2010 and 472/2010 and Cape Nature, permits AAA004-00530-0035.

### Data Deposition

The following information was supplied regarding data availability: The raw data was supplied as a Supplemental Dataset.
