# Supplementary material for: Comparative morphology of the mouthparts of the megadiverse South African monkey beetles (Scarabaeidae: Hopliini): feeding adaptations and guild structure
Source: PeerJ. 2016 Jan 21;4:e1597. doi: 10.7717/peerj.1597 (PMC4727957; doi:10.7717/peerj.1597)
Supplement: Supplemental Information 1 [file peerj-04-1597-s001.docx]

**Appendix A: List of hopliines mouthparts characters**

1. Length – width ratio of the mandible: (0) as long as broad; (1) wider than long.
2. Mola length in relation to overall mandible length: (0) less than one third; (1) more than one third.
3. Mola teeth: (0) absent; present (1).
4. Lacinia mobilis size in relation to mandible: (0) small lobe; (1) large lobe.
5. Lacinia mobilis teeth: (0) absent; (1) present.
6. Lacinia mobilis densely bristled: (0) absent; (1) present.
7. Incisivus: (0) rounded; (1) with cutting edge.
8. Cardo/stipes length - width ratio: (0) stout: less than three times longer than wide; (1) elongated: more than three times longer than wide.
9. Galea shape: (0) short: less than three times longer than wide; (1) elongated: more than three times longer than wide.
10. Galea teeth (0) absent; (1) present.
11. Galea bristles (0) absent; (1) present
12. Ligulae on labium: (0) absent; (1) present.
