# Supplementary material for: Comparative morphology of the mouthparts of the megadiverse South African monkey beetles (Scarabaeidae: Hopliini): feeding adaptations and guild structure
Source: PeerJ. 2016 Jan 21;4:e1597. doi: 10.7717/peerj.1597 (PMC4727957; doi:10.7717/peerj.1597)
Supplement: Supplemental Information 2 [file peerj-04-1597-s002.docx]

| **Appendix C:** Contingency table | | | |
| --- | --- | --- | --- |
| **characters** | **floral tissue** | **pollen** | **nectar** |
| Elongated mandible | 0 | 1 | 2 |
| Prominent mola | 8 | 3 | 2 |
| Mola toothed | 10 | 3 | 3 |
| Lacinia mobilis large | 8 | 4 | 3 |
| Lacinia mobilis toothed | 6 | 2 | 0 |
| Lacinia mobilis densely bristled | 7 | 2 | 3 |
| Incisivus with cutting edge | 4 | 0 | 0 |
| Cardo/stipes elongated | 0 | 0 | 3 |
| Galea elongated | 0 | 0 | 3 |
| Galea toothed | 11 | 2 | 0 |
| Galea bristled | 8 | 4 | 3 |
| Labium with ligulae | 0 | 2 | 3 |
