# Supplementary material for: Comparative morphology of the mouthparts of the megadiverse South African monkey beetles (Scarabaeidae: Hopliini): feeding adaptations and guild structure
Source: PeerJ. 2016 Jan 21;4:e1597. doi: 10.7717/peerj.1597 (PMC4727957; doi:10.7717/peerj.1597)
Supplement: Supplemental Information 3 [file peerj-04-1597-s003.docx]

| **Appendix B:** Character state matrix | | | | | | | | | | | | |
| --- | --- | --- | --- | --- | --- | --- | --- | --- | --- | --- | --- | --- |
| Character | 1 | 2 | 3 | 4 | 5 | 6 | 7 | 8 | 9 | 10 | 11 | 12 |
| Taxa |  |  |  |  |  |  |  |  |  |  |  |  |
| *Anisochelus inornatus* | 0 | 1 | 1 | 0 | 0 | 1 | 0 | 0 | 0 | 1 | 0 | 0 |
| *Anisonyx* *ursus* | 1 | 1 | 1 | 1 | 0 | 1 | 0 | 1 | 1 | 0 | 1 | 1 |
| *Chasme* *decora* | 0 | 1 | 1 | 1 | 1 | 0 | 0 | 0 | 0 | 0 | 1 | 0 |
| *Chasme* sp. | 0 | 1 | 1 | 1 | 1 | 1 | 0 | 0 | 0 | 1 | 1 | 0 |
| *Clania* *glenlyonensis* | 0 | 0 | 1 | 1 | 1 | 1 | 0 | 0 | 0 | 1 | 1 | 0 |
| *Clania macgregori* | 0 | 1 | 1 | 1 | 1 | 1 | 0 | ? | 0 | 1 | 1 | 0 |
| *Congella* sp. | 0 | 1 | 0 | 1 | 0 | 1 | 1 | 0 | 0 | 1 | 0 | 0 |
| *Dolichiomicroscelis gracilis* | 0 | 0 | 1 | 1 | 0 | 0 | 0 | ? | 0 | 1 | 1 | 0 |
| *Heterochelus pickeri* | 1 | 0 | 0 | 1 | 0 | 1 | 0 | 0 | 0 | 1 | 1 | 0 |
| *Kubousa gentilis* | ? | ? | 1 | 0 | 0 | 0 | 1 | ? | 0 | 1 | 1 | 0 |
| *Lepisia* *ornatissima* | 0 | 1 | 1 | 1 | 1 | 0 | 0 | 0 | 0 | 1 | 1 | 0 |
| *Lepisia rupicola* | 0 | 1 | 1 | 1 | 1 | ? | 1 | 0 | 0 | 1 | 1 | 0 |
| *Lepithrix* sp. | 0 | 1 | 1 | 1 | 1 | 0 | 0 | 0 | 0 | 0 | 1 | 1 |
| *Mauromecistoplia* *nieuwoudtvillensis* | 0 | 1 | 1 | 0 | 0 | 1 | 1 | 0 | 0 | 1 | 0 | 0 |
| *Pachycnema* *calcarata* | 1 | 0 | 1 | 1 | 0 | 1 | 0 | 1 | 1 | 0 | 1 | 1 |
| *Pachycnema* *crassipes* | 0 | 1 | 1 | 1 | 0 | 1 | 0 | 1 | 1 | 0 | 1 | 1 |
| *Pachycnema* *flavolineata* | 0 | 1 | 1 | 1 | 0 | 1 | 0 | 0 | 0 | 1 | 1 | 1 |
| *Scelophysa* *scheffoldi* | 0 | 1 | 1 | 1 | 1 | 1 | 0 | ? | 0 | 1 | 1 | 0 |
